# Decoding the Myeloid-Derived Suppressor Cells in Lymphoid Malignancies

**DOI:** 10.3390/jcm10163462

**Published:** 2021-08-04

**Authors:** Iosif Papafragkos, Efrosyni Markaki, Christina Kalpadakis, Panayotis Verginis

**Affiliations:** 1 Laboratory of Immune Regulation and Tolerance, Division of Basic Sciences, Medical School, University of Crete, 71003 Heraklion, Greece; iosif.pap96@gmail.com (I.P.); effrosynimarkaki@gmail.com (E.M.); 2 Laboratory of Haematology, Division of Laboratory Medicine, Medical School, University of Crete, 71003 Heraklion, Greece; 3 Department of Laboratory Haematology, University Hospital of Heraklion, 71500 Heraklion, Greece

**Keywords:** Myeloid-derived suppressor cells, lymphomas, immune regulation

## Abstract

Myeloid-derived suppressor cells (MDSCs) are immature myeloid precursors which emerged as a potent regulator of the immune system, exerting suppressive properties in diverse disease settings. In regards to cancer, MDSCs have an established role in solid tumors; however, their contribution to immune regulation during hematologic malignancies and particularly in lymphomas remains ill-defined. Herein focused on lymphoma, we discuss the literature on MDSC cells in all histologic types, and we also refer to lessons learned by animal models of lymphoma. Furthermore, we elaborate on future directions and unmet needs and challenges in the MDSC field related to lymphoma malignancies which may shed light on the complex nature of the immune system in malignancies.

## 1. Introduction

Myeloid cells have been reported for their suppressive ability around 40 years ago [1,2]. However, the nomenclature “Myeloid-derived suppressor cells” (MDSCs) was first established in 2007 [3,4]. Up to date, MDSCs are characterized as a heterogeneous population of immature cells of myeloid origin. Despite the similarities with neutrophils and monocytes, MDSCs are distinct from terminally differentiated mature myeloid cells, mainly focused on their potent immunosuppressive activity as well as the metabolic and transcriptomic profile [5]. MDSCs have emerged as a unique regulator of the immune system and have been demonstrated to heavily expand and to be implicated not only in cancer pathogenesis, growth, and metastasis [6] but also in various aspects of immune regulation, including chronic inflammation, infectious diseases, autoimmune diseases, trauma, graft versus host disease and so on [7,8,9,10]. 

## 2. Phenotypic Characterization of MDSCs 

The phenotypic and morphologic heterogeneity impede the precise characterization of MDSCs. However, the phenotypic characterization of MDSCs in mice has been established and is well-defined [11], based on the expression of the myeloid-cell lineage differentiation antigen Gr-1 [12,13] and CD11b [14] (αM-integrin) markers. Extensive research on MDSC biology supports their morphological heterogeneity, subdividing them into two functionally distinct subsets named the monocytic-MDSCs (M-MDSCs), which resemble monocytes and expressing the Ly-6C (CD11b^+^Ly-6G^–^Ly-6C^hi^), and granulocytic or polymorphonuclear MDSCs (PMN/G-MDSCs), which are characterized based on the Ly-6G expression MDSCs (CD11b^+^Ly-6G^+^Ly-6C^low^) [15].

In contrast, human MDSCs are not clearly defined due to their early-stage cell nature and their difficulty to be phenotypically and mechanistically defined. Both of MDCSCs’ subgroups are isolated from low-density Ficoll-gradient fraction of peripheral blood mononuclear cells (PBMCs), a mean which makes their distinction an easy task, as neutrophils show a similar phenotype and are found in high-density fraction [11]. Thus, as human PMN-MDSCs are defined the CD11b^+^CD14^–^CD15^+^CD33^+^ or CD11b^+^CD14^–^CD66b^+^CD33^+^ cells [11,16], with CD15 and CD66b being human granulocyte activation markers. Regarding the human M-MDSCs, these cells are characterized by the CD11b^+^CD14^+^CD15^–^CD33^+^HLA-DR^–/lo^ phenotype [11] and are distinguished from human monocytes by low expression or absence of HLA-DR molecules. Furthermore, “early-stage MDSCs” (eMDSCs) have been described, defined as an immature MDSC population and characterized as Lin^−^HLA-DR^−^CD33^+^ cells, where Lin includes CD3, CD14, CD15, CD19, and CD56 [11,17]. However, the mouse equivalent is yet to be identified. Delineation of new markers that will specifically characterize MDSCs and their subgroups in humans is an unmet need, and their identification holds significant clinical importance since it will expand our knowledge in MDSC biology and may help the stratification of patients and guide personalized medicine decisions. In relation to this, the identification of lectin-type oxidized LDL receptor 1 (LOX-1) [18,19,20,21,22,23] as a specific surface marker of human PMN-MDSCs allowed their gradient-independent purification and characterization in cancer patients. Such knowledge may pave the way for the identification of novel MDSC targets and may lead to the design of future immunotherapeutic approaches. 

## 3. Expansion and Activation

The mechanisms that govern the generation, expansion, and activation of MDSCs are complex and under current investigation. MDSCs are generated under pathological conditions through emergency myelopoiesis, and their expansion is mediated by several factors in the bone marrow (BM) or spleen before they migrate to the periphery. Several factors such as granulocyte-macrophage colony-stimulating factor (GM-CSF) [24], granulocyte colony-stimulating factor (G-CSF) [25], macrophage colony-stimulating factor (M-CSF) [26], stem cell factor (SCF) [27], vascular endothelial growth factor (VEGF) [13], and proinflammatory factors, such as interleukin-6 (IL-6) [28], alarmins S100A9 and S100A8 [29], and prostaglandin E2 (PGE2) [30,31] have been shown to mediate this process. These induce signaling pathways, which are controlled by transcription factors and regulators, such as signal transducer and activator of transcription (STAT)-3, STAT5, Interferon regulatory factor 8 (IRF8), and C/EBP-β (reviewed in [32,33,34]). In addition, signaling pathways of NOTCH, adenosine receptor A2b, cytoplasmic receptor NLRP3, and retinoblastoma protein 1 (RB1) are also involved in MDSC expansion (reviewed in [32,33]). Induction of MDSC suppressive activity, except signals that promote their expansion, also requires signals for their activation, including interferon-γ (IFN-γ), IL-1β, IL-4, IL-6, IL-13, tumor necrosis factor-α (TNF-α), toll-like receptors (TLRs) ligands, and transforming growth factor-β (TGFβ) (reviewed in [33,35,36]). Interestingly, recent studies suggest that M-MDSCs are induced by the reprogramming of monocytes, while PMN-MDSCs may represent an activation stage of neutrophils generated from activated immature or mature granulocytes [37].

## 4. Recruitment

The accumulation and recruitment of MDSCs in the microenvironment in different inflammatory situations have been demonstrated to be induced by a variety of factors. Chemokines are the main soluble mediators that are involved in this process. For example, CCL2- and CCL5-mediated recruitment of MDSCs have been reported at the inflammatory tumor microenvironment (TME) [38], as increased levels of intratumoral CCL2 were found in patients with colitis-associated colorectal cancer (CRC), adenocarcinomas, and adenomas [39], whereas increased expression of CCR2 (receptor of CCL2) was found on isolated MDSCs from ovarian, breast, or gastric cancer patients [40]. In addition, CCR2-deficient MDSCs and the deletion of CCL2 reduced the recruitment of MDSCs into tumor sites of murine tumor models [39,40,41]. Moreover, CCL5 chemokine expression has been correlated with advanced breast cancer in human patients [42] and was reported to promote mammary tumor growth in mice, whereas the deficiency of this chemokine resulted in the aberrant generation of MDSCs in the bone marrow, with the impaired immunosuppressive ability [43]. In the same line, other chemokines, including CCL7 [44], CCL15 [45], CCL26 [46], CXCL8 (also known as IL-8) [47], and CXCL12 [48], have been demonstrated to mediate mobilization of MDSCs in TME.

## 5. Mechanisms of Function

MDSCs employ a variety of mechanisms, such as cell-surface receptors and/or the release of soluble factors, to suppress immune responses with M-MDSCs to be a more potent suppressor subset compared to PMN-MDSCs, which have a relatively modest suppressive activity [49,50].

The main mechanisms through which MDSCs exert their immunosuppressive function are briefly outlined below.

### 5.1. Deprivation of Essential Amino Acids

The metabolism of L-arginine is one of the well-characterized mechanisms of MDSC immunosuppressive activity. L-arginine is a substrate for the inducible Nitric Oxide Synthase (iNOS) and Arginase 1 (ARG-1), both of which are highly expressed in MDSCs and involved in lymphocyte suppression [51,52] since the deprivation of L-arginine leads to T-cell dysfunction [53,54]. In addition, induced iNOS production from MDSCs generates nitric oxide (NO), which is implicated in the attenuation of MHC class II expression in macrophages [55] and the induction of T-cell apoptosis [56,57,58]. This mechanism has been extensively studied in solid tumors but also in hematological malignancies. Thus, MDSCs in lymphoma [59,60,61,62] and multiple myeloma [63,64] patients were correlated with an upregulated gene expression of ARG-1 and iNOS, and increased expression of ARG-1 in PMN-MDSCs was associated with the disease progression and the resistance to therapy [65]. Besides L-arginine, competition for extracellular cysteine between APCs and MDSCs resulted in the deprivation of cysteine and thus the inhibition of T-cell activation [66]. Moreover, indolamine 2,3-deoxygenase (IDO)-dependent tryptophan catabolism is considered a mechanism in MDSC-mediated suppression. This generated the notion of targeting IDO1 for therapeutic purposes (Reviewed in [67,68]). However, IDO1 inhibitor monotherapies did not show the expected efficacy, whereas the combination with immunotherapeutic approaches showed negative results in phase-III clinical trials [67], indicating that further studies are needed for the effective targeting of IDO in cancer.

### 5.2. Reactive Oxygen Species (ROS) Production

Another important factor that is induced in activated MDSCs and contributes to their immunosuppressive activity is ROS and peroxynitrite (PNT) production. Up-regulation of ROS in MDSCs is observed in both tumor-bearing mice and cancer patients with solid tumors [69,70,71,72] mediated by the increased activity of NADPH oxidase (NOX2). Moreover, increased MDSC-mediated production of ROS is also observed in the lymphoma murine model [15] and patients with mycosis fungoides and Sézary syndrome with stage IB T-cell malignancies [73]. ROS production, together with PNT, has been accused of T-cell unresponsiveness and tolerance, thus targeting their production seems to be a promising therapeutic intervention, which would enhance the effects of immunotherapeutic approaches and help to induce antitumor immune responses (reviewed in [74]). 

### 5.3. Obstruction of Lymphocyte Trafficking

MDSCs in the context of their immunosuppressive nature downregulate the expression of L-selectin (CD62 ligand), a key homing receptor on T-cells, as reported by the in vitro co-culture of tumor-induced MDSCs with naïve T-cells [75,76]. The downregulation of L-selectin has been demonstrated to be associated with the expression of ADAM17 (disintegrin and metalloproteinase 17) on MDSCs [77]. 

### 5.4. Induction of Immunosuppressive Cell Population and NK Cell Anergy

Finally, MDSCs promote the generation of Treg through the secretion of TGF-β and IL-10 in solid tumor murine models [78]. Furthermore, CLL-induced MDSCs have been shown to promote Treg induction in an IDO-dependent manner [79]. In addition to MDSC-mediated Treg induction, MDSCs are also capable of increasing some M2-like characteristics of macrophages, thus inducing an immunosuppressive phenotype of them and promoting the growth of solid tumors [80]. MDSCs through TGF-β suppressed NK function by the inhibition of their IFN-γ production, NKG2D expression, and their cytotoxic activity in vitro and in an in vivo hepatic model [81].

PMN-MDSCs and M-MDSCs have unique features through which they mediate their suppressive function. More specifically, PMN-MDSCs-mediated immunosuppression often requires ARG-1, ROS, and PNT, whereas M-MDSCs suppress antitumor responses through NO, the secretion of IL-10 and TGF-β immunosuppressive cytokines, and the expression of inhibitory surface molecules, such as PDL-1. Due to MDSC heterogeneity, targeting them is not an easy task. In addition, recent studies revealed mechanisms that mediate the reprogramming of myeloid suppressor cells and enhance the anti-tumor immune responses [82,83]. Thus, the identification of novel mechanisms of MDSC-mediated suppression, which characterize each subgroup, are required. This will offer insights in MDSC biology but also, together with the discovery of specific markers that will exclusively characterize these cells, could pave the way for the design of innovative therapeutic interventions that will further improve the already established. 

## 6. MDSCs in Hematological Malignancies

Numerous studies have established the role of MDSCs in several hematological malignancies, such as multiple myeloma (MM), acute myeloid leukemia (AML), acute promyelocytic leukemia (APL), B-cell acute lymphoblastic leukemia (B-ALL) myelodysplastic syndromes (MDS), and in chronic myeloid leukemia (CML), as extensively reviewed elsewhere [84]. A common denominator in these diseases is that frequencies of MDSCs are significantly enriched either in blood, bone marrow, or tumor sites and often associated with high tumor burden, adverse clinical features, and outcome. Herein, we focus on lymphomas, a group of blood malignancies of lymphocytes, and we discuss the lessons learned from MDSC-mediated suppression in mouse models and in individuals with lymphomas. 

## 7. MDSCs in Mouse Models of Lymphoma

For the first time in 2008, Serafini et al. showed that MDSCs can also contribute to antigen-specific T-cell tolerance in a systemic hematological malignancy using a murine A20 B-cell lymphoma model. More specifically, 28 days after intravenous injection of CD45.2 A20-HA-GFP cancer cells into BALB/c mice, they found a cell population with a phenotype consistent with other murine MDSCs described in solid tumors, which was characterized by the high expression of CD11b, low expression of MHC class I and II molecules, and expression of Gr1, F4/80, and IL-4Ra. Through further functional analysis, they confirmed that these cells were MDSCs, and by performing both in vivo and in vitro experiments, they showed that these cells can uptake and process tumor-associated antigens and promote the expansion of a preexisting Treg pool exclusively via arginase-1, which, in turn, induced antigen specific T-cell anergy and was responsible for the immunosuppressive state associated with an increasing tumor burden in lymphoma-bearing mice [85].

Until now, several studies in B-cell lymphoma patients have shown increased MDSC frequencies with enhanced immunosuppressive potential, while others have highlighted the role of microRNAs (miRNAs) in the regulation of their differentiation, maturation, and function. Zhen Xu et al., using a B-cell lymphoma mouse model, observed that only miR-30a and no other members of the same family were increased in both the bone marrow and spleens of tumor-bearing mice. Thus, they decided to explore its role in the regulation of MDSC differentiation and function. Interestingly, they demonstrated that miR-30a could promote their maturation and affect the expression of their main suppressive mediators, including those of Arg-1, IL-10 and ROS. Furthermore, they showed that SOCS3, which is a regulator of the JAK2/STAT3 pathway that plays a critical role in the regulation of MDSC expansion and function, is a direct target of miR-30a. As a consequence, they found that miR-30a downregulated the JAK2/STAT3 signaling pathway by reducing the expression of SOCS3 and thus promoted their differentiation and suppressive activity. Finally, by performing transfection with miR-30a mimics, they observed elevated frequencies of fully differentiated MDSCs with enhanced suppressive potential, suggesting that targeting miR-30a could reverse the deleterious effect of MDSCs in antitumor immunity [86].

In addition, several lymphoma and leukemia studies have examined the serum levels of adiponectin (APN) and have revealed an increase in adult and childhood non-Hodgkin lymphoma patients, which was correlated with poor prognosis. However, few molecular studies have been conducted on the role of APN in lymphoma development and progression. In 2013, Sora Han et al. studied the role of APN in tumor growth and in the function of MDSCs by using EL4 lymphoma-bearing APN knock-out (APNKO) mice. Interestingly, they found that tumor-bearing APNKO mice had a reduced accumulation of MDSCs in their spleen, exhibited a decreased G-CSF secretion which was correlated with reduced MDSC differentiation as well as that APN facilitated their differentiation and function by upregulating the iNOS transcription levels [87]. 

Moreover, in 2016, M. Abedi-Valugerdi et al., employing a murine lymphoma model, observed that mice with established EL4-luc2 tumors exhibited a marked infiltration of MDSCs in their spleen, as well as an increased frequency of circulating neutrophils with ring-shaped nuclei, a phenomenon known as tumor-induced neutrophilia, suggesting that in tumor-bearing mice, the process of myelopoiesis is significantly enhanced during tumor development. Importantly, they also determined that more than 90% of the circulating neutrophils of tumor-bearing mice represent MDSCs, and 75% of them were PMN-MDSCs. These findings support the hypothesis that tumors alter normal myelopoiesis and favor the generation of MDSCs in order to evade the immune response. In addition, they assessed the sensitivity of tumor-associated neutrophils to low-dose myelosuppressive drugs 5-FU and BU, and they found that 5-FU and Treo but not BU induced tumor regression by eliminating MDSCs at both their immature and mature stage [88]. The following year, the same group of scientists investigated the effects of 5-FU on MDSCs at two different stages of tumor development, an early and a more advanced. They found that when mice with advanced tumors were treated with a single-low dose of Flu, they exhibited elevated numbers of splenic MDSCs, as well as increased MDSC-mediated immunosuppression, which potentiated tumor growth. On the other hand, neither the tumor growth nor the number of splenic MDSCs was significantly affected in mice with palpable tumors following the same treatment. In contrast, the administration of a low-dose 5-FU, independently of the tumor stage, resulted in tumor regression that coincided with a significant reduction in the number of blood neutrophils and splenic MDSCs as well as a marked attenuation of their relative immunosuppressive effects [89]. In line with the above studies, Pilot et al., using a murine T-cell lymphoma model recently unveiled the effect of heat shock (HS) or HSP70 deficiency on the 5-FU-mediated activation of caspase 1/IL-1β in MDSCs and its following consequences on tumor progression. More analytically, they showed that HS is able to dampen caspase-1 activation without affecting 5-FU-induced MDSC death, which results in the promotion of antitumor immunity and the inhibition of tumor growth. Conversely, they revealed that the absence of HSP70 in HSP70^−/−^ tumor-bearing mice increased caspase-1 activation in MDSCs and caused tumor regrowth, while the HS was able to increase the survival of these mice after 5-FU treatment, suggesting that in the absence of HSP70, hyperthermia may use other compensatory pathways. Therefore, this work proposed an innovated non-chemical strategy to inhibit the caspase-1/IL-1β activation within MDSCs in order to improve 5-FU-based chemotherapeutic treatment efficiency using hyperthermia [90].

Furthermore, in 2014, Hong Qin et al. adapted a competitive peptide phage display platform, and they created peptide-Fc fusion proteins named as peptibodies, which were able to completely deplete blood, splenic, and intratumoral MDSCs in lymphoma-bearing mice after their intravenous administration. Importantly, these peptibodies did not show any off-target activity as the populations of DCs, T, B, NK cells, or Gr1^+^ CD11b^+^ immature myeloid precursor cells in the bone marrow were unaffected. Additionally, their superior therapeutic effects were highlighted by their ability to deplete both granulocytic and monocytic MDSC subsets and to promote tumor regression in vivo over the available Gr1 specific antibodies, which primarily depleted only the granulocytic subset [91]. Moreover, the same year, another study that examined whether lenalidomide could act as an immune adjuvant to enhance the therapeutic potency of cancer vaccines in mice with A20 lymphoma revealed that lenalidomide could promote tumor regression and improve immune suppression by reducing the number of systemic MDSCs in tumor-bearing but not naive mice. Nevertheless, its exact effect on the regulation of their function, as well as its potent direct MDSC cytotoxicity, remains to be unveiled [92]. 

In 2020, Fei Lu et al. examined the contribution of NLRP3 inflammasome in the modulation of immune homeostasis during lymphomagenesis, along with its correlation with PD-L1 expression levels and the effectiveness of anti-PDL1 immunotherapy in combination with NLRP3 blockade. So as to achieve that, they analyzed peripheral blood samples isolated either from Diffuse Large B-Cell Lymphoma (DLBCL) patients or from healthy individuals, and they observed that DLBCL patients were characterized by an immunosuppressive state associated with a high expression of PDL1 in the tumor tissues together with elevated frequencies of MDSCs. In order to further explore the role of NLRP3 inflammasome in B-cell lymphoma progression, they employed a murine lymphoma model by injecting subcutaneously A20 cancer cells into 4-week-old female BALB/C mice and treating them with the NLRP3-inhibitor MCC950 or with placebo. Significantly, they found that the NLRP3 blockade resulted in delayed tumor progression, and so, to further determine whether this attenuation in tumor growth derived from the reversal of immunosuppression, they analyzed the frequencies of immune cell populations in lymphoma-bearing mice. Interestingly, they found that MDSCs frequencies, as well as the frequencies of other immunosuppressive cells, such as TAMs and Tregs, were decreased in tumor tissues and spleens of treated mice. Furthermore, in order to examine the influence of NLRP3 inflammasome on the efficacy of ICB, they used three groups of lymphoma-bearing mice: the first was treated with the MCC950 inhibitor, the second with the anti-PDL1 antibody, and the latter with combination therapy. What they observed is that, in contrast to NLRP3 inflammasome blockade, both anti-PDL1 and combination treatment markedly increased the frequencies of tumor-infiltrating MDSCs without affecting the Treg population, indicating that the PDL1 blockade partly revoked the immune-stimulatory function of MCC950 in TME [93].

Given the fact that IL-35 plays an important role in inducing the accumulation of MDSCs in TME and promoting angiogenesis, Wang et al. sought to reveal the contribution of this immunosuppressive cytokine in the pathogenesis of DLBCL. For this reason, they injected NOD-SCID mice with Ly8 cells subcutaneously into their flank in order to develop Ly8 DLBCL tumors, and 7 days after tumor inoculation, they treated them with anti-IL35 or IgG2b antibody once weekly for 2 weeks. At day 23, after tumor induction, they sacrificed them, collected blood, and analyzed their M-MDSC populations. Importantly, they found that tumor-bearing mice treated with neutralizing anti-IL35 antibodies had a reduced accumulation of M-MDSCs, indicating that anti-IL-35 treatment blocked M-MDSC expansion, creating expectations for their possible future use as a new therapeutic strategy for DLBCL patients [94].

Conclusively, mouse models provide important knowledge on MDSCs’ function in lymphomas. However, various mechanisms still remain to be validated in human counterparts and especially in the field of lymphomas, which constitute a highly heterogeneous group of hematologic malignancies.

## 8. MDSCs in Lymphoid Malignancies

**Figure 1:**
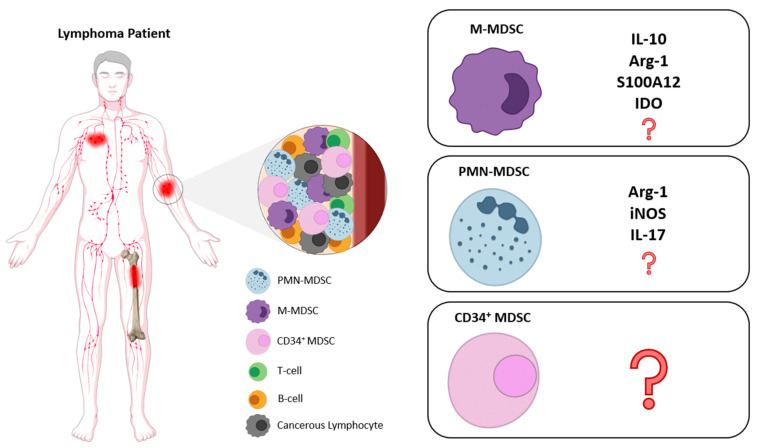
MDSC subsets and suppression mechanisms in lymphoid malignancies. The main immune cell subsets and malignant cells that circulate in the blood of lymphoma patients are illustrated (**left side**). The MDSC subsets and the respective mechanisms that have been described for MDSC-mediated suppression of anti-tumor immunity in different types of lymphomas are shown (**right side**). Question marks indicate unknown mechanisms via which MDSCs may exert their function in lymphoma malignancies. MDSCs: Myeloid-derived suppressor cells; PMN-MDSCs: polymorphonuclear MDSCs; M-MDSCs: monocytic-MDSCs; IL-10: Interleukin-10; Arg-1: Arginase 1; S100A12: S100 calcium-binding protein A12; IDO: indoleamine 2,3-dioxygenase; iNOS: inducible Nitric Oxide Synthase; IL-17: Interleukin-17.

The assessment of MDSCs in lymphoma patients remains limited and mostly focused on Diffuse Large B-cell Lymphomas (DLBCL) and Hodgkin’s Lymphoma (HL) because few studies have been conducted in other histological types of non-Hodgkin’s Lymphoma (NHL), which do not permit drawing firm conclusions for the predominant phenotype and functional role of MDSCs in all NHL categories. Therefore, considering the established role of MDSCs in the suppression of immune responses, it is important to shed light on their involvement in lymphoma development and progression as well as during therapeutic interventions in different types of lymphoma. Below, we review the literature on the role of MDSCs and subsets in lymphoid malignancies and discuss the proposed mechanism of action. In Figure 1 and Table 1, we summarize the main findings on MDSC contribution in lymphoid malignancies in terms of phenotype and characterization, mechanisms of action as well as clinical implications. 

### 8.1. MDSCs in B-Cell Lymphomas

DLBCL represents the best-studied lymphoma category regarding the phenotype and role of MDSCs. DLBCL is the most common subtype of NHL, representing 30–40% of all cases. During the last years, there has been great progress in the understanding of the biology and the more accurate classification of DLBCL-NOS (not otherwise specified), which have been divided into two broad categories: those derived from germinal center (GCB) and those derived from activated B-cells (ABC). The incorporation of Rituximab into the standard CHOP chemotherapy (R-CHOP-21) has revolutionized the treatment and outcome of DLBCL patients. In an initial attempt to characterize the MDSC population in DLBCL patients, Tadmor et al. focused on the observation that lymphoma patients often exhibit monocytosis, and thus they sought to correlate the contribution of M-MDSCs in this phenomenon [60]. To this end, in 91 newly diagnosed DLBCL patients, M-MDSCs characterized as CD14^+^HLADR^low^ in the CD45^+^ fraction of PBMCs, and their frequencies were analyzed. Patients with DLBCL had an average increase in M-MDSCs counts at diagnosis compared to controls, and interestingly, M-MDSC levels returned to normal after achieving remission. 

In one of the largest studies published so far in the literature, it included 144 newly diagnosed DLBCL patients [95]. Circulating M-MDSCs and monocyte levels were analyzed at diagnosis and correlated with the Revised-International Prognostic Index (R-IPI) and the histologic subtype (Germinal Center Phenotype (GCB) vs. non-GCB). In total, 63 patients belonged to the GCB category and 81 to the non-GCB. M-MDSCs’ (CD14^+^HLADR^low/–^) frequencies were significantly higher in DLBCL patients compared to healthy controls in all risk groups according to R-IPI. When M-MDSCs were analyzed according to the histologic subtype, significant differences were found between GCB and non-GCB in both poor and very good groups, whereas no difference was found in the good group. Significant differences were also observed among the risk groups according to R-IPI both in GCB as well as in the non-GCB patients. In addition, a positive correlation was found between monocyte levels and M-MDSCs levels [95]. A follow-up study evaluated the prognostic significance of circulating M-MDSCs in DLBCL patients after they were treated with the immunochemotherapeutic combination: R-CHOP (Rituximab plus cyclophosphamide, doxorubicin, vincristine and prezolon) [96]. Monocytes and M-MDSCs significantly decreased after receiving R-CHOP in all prognostic groups based on R-IPI. Despite the thorough analyses of these studies on M-MDSCs in a large cohort of DLBCL patients, the assessment of PMN-MDSCs levels, as well as their correlation with CPG phenotype, was not reported.

Analyses of PMN-MDSCs were also excluded from a recent study by Wang et al., in which 103 DLBCL patients (65 newly diagnosed, 12 in relapse, and 26 in remission) and 30 healthy controls were examined for M-MDSCs’ (CD14^+^HLADR^−/low^) frequencies in their peripheral blood [94]. Notably, a significantly increased frequency of M-MDSCs was found in the 65 newly diagnosed as well as relapsed DLBCL patients compared to patients in remission and healthy controls. M-MDSCs levels were also correlated with the disease stage (III/VI vs. I/II), LDH levels, and IPI, while no correlation was found between MDSCs and the histologic subtype (GCB vs. non-GCB). In addition, and in accordance with the study by Wu et al. [96], the frequency of M-MDSCs was significantly decreased after R-CHOP therapy. The negative correlation between the overall survival (OS) and the frequency of M-MDSCs was further validated. Based on the median value frequency of M-MDSCs (25.4%), DLBCL patients were divided into two groups: low (*n* = 39) with M-MDSCs levels ≤ 25.4% and the high group (*n* = 26) with M-MDSCs levels > 25.4%. The OS of DLBCL patients with low M-MDSC levels was significantly longer than those with high M-MDSC levels (*p* < 0.01) [94]. In multivariate analysis, IPI and MDSCs levels were independent prognostic factors for OS. Furthermore, the increased levels of the immunosuppressive cytokine IL-35 in the periphery of DLBCL patients initiated the experiments on the role of IL-35 in M-MDSC expansion in NOD-SCID humanized animals, as described above. Overall, this study provides insight into the mechanisms that may lead to M-MDSC expansion in patients with lymphoma and highlights the necessity of identification of additional molecules that drive M-MDSC accumulation and expansion and enforce their suppressive activity.

In line with this, a study by Azzaoui et al. in newly diagnosed DLBCL patients demonstrated significantly increased numbers of circulating PMN-MDSCs (Lin^−^HLADR^−^CD33^+^CD11b^+^) and M-MDSCs’ (CD14^+^HLADR^low^) frequencies compared to healthy controls [97]. Using gene expression profiling (GEP), a myeloid suppressive signature was identified in DLBCL peripheral blood, which consisted of an upregulated expression of MDSC-related genes, such as ARG-1, S100A12, and S100A8. The increased expression of these genes was associated with the decreased event-free survival (EFS) compared to patients with low expression of these genes. Of interest, only the M-MDSCs number was correlated with the high-risk features and poor outcome, and a significant correlation of M-MDSCs with the number of circulating Tregs was reported. In addition, M-MDSCs suppressed autologous CD8^+^ T-cell proliferation upon polyclonal activation, compared to those from healthy donors, suggesting a specific immunosuppressive function of M-MDSCs in DLBCL patients [97]. This was further confirmed since depletion of the CD14^+^ monocytes restored T-cell proliferation. In an effort to address the mechanism of M-MDSC-mediated suppression, they tested arginase and IDO inhibitors since both molecules were increased in the periphery of DLBCL patients. However, neither inhibitor managed to reverse the suppression of T-cell proliferation, suggesting that other mechanisms operate in M-MDSCs to exert their suppressive function in this particular patient cohort. Based on gene and protein expression analyses, IL-10, S100A12, and CD274/PD-L1 were found to be increased in DLBCL patients and neutralization of these molecules resulted in an increase in both CD4 and CD8 T-cell proliferation. Therefore, the authors concluded that MDSC suppressive activity was mediated by the release of IL-10 and S100A12 and the increased expression of programmed death-ligand 1 (PD-L1) by lymphoma cells.

Besides those studies in DLBCL patients, other reports elaborate on the MDSC frequencies and function in various histologic types of B-cell lymphomas. Thus, Lin et al. reported on 40 B-cell lymphoma cases, 36 of which were relapsed/refractory and only 4 newly diagnosed [61]. The histologic subtypes included were: DLBCL N = 18, Follicular lymphoma (FL) N = 14, Mantle cell lymphoma (MCL) N = 4, Small lymphocytic lymphoma (SLL) N = 2, Mucosa-Associated Lymphoid Tissue (MALT) lymphoma N = 1, and Lymphoplasmacytic (LPL) N = 1. Specifically, they described the presence of a suppressive monocytic population in the peripheral blood of patients characterized as CD14^+^HLA-DR^low/−^ and expressed low levels of the tumor necrosis factor α receptor II (CD120b) corresponding to the M-MDSC subset. Of interest, the depletion of monocytes increased the proliferation of peripheral blood T-cells, indicating their immunosuppressive function. The supplementation of exogenous arginine partially overcame monocyte-mediated suppression, suggesting that arginine metabolism may contribute to their suppressive activity and further characterization of NHL monocytes revealed impaired STAT1 phosphorylation and reduced type I IFN production. Importantly, patients with increased M-MDSCs had more aggressive disease: higher disease stage, more aggressive pathology, and faster rate of disease progression. An interesting observation of this study was that the assessment of frequencies of Lin^−^CD33^+^HLA-DR^−^ cells corresponding to PMN-MDSCs as well as circulating Tregs, which revealed no significant differences between NHL patients and healthy controls [61]. Whether this applies to various NHL cohorts and to different stages of diseases remains to be investigated.

In a similar fashion, in a study by Khalifa et al., 42 lymphoma patients were evaluated for the presence of M-MDSCs (CD14^+^HLA-DR^low/–^) in peripheral blood, with 26 newly diagnosed and 16 relapsed/refractory [62]. Among them, 21 were DLBCL, 16 had Follicular Lymphoma (FL), and 5 had indolent B-NHL. Following the flow cytometry analysis, frequencies of M-MDSCs were significantly increased in lymphoma patients compared to healthy individuals, and the elevated M-MDSC levels correlated with an advanced stage of disease (III/IV), aggressive histology (DLBCL vs. other low-grade B-NHLs), and relapsed/refractory disease, which was also correlated with increased plasma levels of Arg-1. However, the immunosuppressive properties of M-MDSCs, as well as whether Arg-1 contributes to their functional properties, were not addressed in this study. 

One year later, Xiu et al. aimed to shed more light on the underlying mechanism facilitating the development of CD14^+^HLA-DR^low/−^ monocytes (M-MDSCs) in lymphoma [98]. For this, they enrolled 22 newly diagnosed lymphoma patients: 6 DLBCL, 7 FL, 4 MCL, and 5 Marginal Zone Lymphoma (MZL) and found that circulating M-MDSCs were elevated in newly diagnosed patients with B-NHL compared to healthy controls and also correlated with a higher IPI. Interestingly, driven by the increased IL-10 serum levels in lymphoma patients, they sought to address whether IL-10 may trigger M-MDSC development. Indeed, in vitro exposure of monocytes to IL-10-rich supernatants obtained from lymphoma cells induced downregulation of HLADR expression, resulting in the phenotype of M-MDSCs with immunosuppressive function over T-cell proliferation [98]. 

Overall, studies on B-cell lymphomas concur on the increased frequencies of M-MDSCs (CD14^+^HLA-DR^low/−^) in the periphery of patients compared to healthy individuals and provided evidence for their immunosuppressive mechanisms. Regarding the role of the PMN-MDSC cells in NHL patients, knowledge is very limited, and thus, more data should be generated to address the role of this MDSC subset not only in terms of frequencies but also function and generation. Towards this, the inclusion of LOX-1 marker in the PMN-MDSCs phenotypic panel may allow their better characterization and functional assessment in lymphoma patients.

### 8.2. MDSCs in Hodgkin Lymphoma (HL)

HL is a lymphoid tumor with characteristic multinucleated giant cells termed Reed-Sternberg (RS) within an inflammatory milieu. The neoplastic cells are of B-cell lineage (PAX5+) in virtually all cases. HL usually affects young individuals with a localized nodal disease and indolent clinical behavior. Cure rates are in excess of 80–90% with chemotherapy. For relapsed/refractory disease, the recent finding of PD-L1 expression warrants the use of checkpoint inhibitor immunotherapy.

To address the role of MDSCs in Hodgkin’s Lymphoma (HL), Romano et al. examined 60 newly diagnosed patients and aimed to correlate MDSC frequencies with disease phenotype and therapeutic outcome [99]. For this purpose, they divided MDSCs into three subtypes based on the surface phenotype, named monocytic (CD14^+^CD11b^+^HLADR^−^), granulocytic (CD33^+^CD11b^+^CD14^−^HLA-DR^−^), and a highly immature MDSC subset characterized by the expression of CD34^+^, termed CD34^+^MDSCs. Whole blood was used and compared with 25 age-matched healthy controls (HC). Overall, the median number of MDSCs was higher in HL compared to HC for both types of MDSCs, and importantly, MDSC levels were higher in non-responders compared to responders suggesting that MDSCs may be considered a biomarker for prediction of response. In this line, a cut-off level of 0.0045 × 10^9^/l for CD34^+^MDSC displayed a strong prognostic significance for PFS with a specificity and sensitivity of 92% and 89%, respectively, suggesting that CD34^+^MDSCs levels could serve as a prognostic biomarker in HL patients. 

Although in several studies with lymphoma patients the role of PMN-MDSCs has been ignored, Marini et al. addressed the role of PMN-MDSCs in both Hodgkin and non- Hodgkin lymphoma patients [100]. Specifically, 124 patients (31 with HL, 62 with high grade, and 31 with indolent B-cell lymphoma) were analyzed and compared to 48 healthy donors. PMN-MDSCs were recognized by the expression of CD66b^+^CD33^dim^HLA-DR^−^ and by their ability to suppress T-cells. PMN-MDSC was increased in PBMCs from lymphoma patients compared to healthy donors and correlated with prognostic index scores, freedom from progression and disease status. Interestingly, this is one of the few studies that propose a potential suppressive role of PMN-MDSCs in lymphoma patients, and therefore, further investigation is required towards the delineation of PMN-MDSC-mediated immune suppression in lymphomas. 

In a similar fashion, Amini et al. investigated different immune regulatory cell subsets in the peripheral blood of lymphoma patients at diagnosis in relation to clinical characteristics and treatment outcome [101]. A total of 43 newly diagnosed lymphomas were included in the study: 24 high-grade B-cell lymphomas and 19 HL along with 15 healthy controls. An altered immune profile was ascertained in the PB of lymphoma patients characterized by a lower percentage of regulatory NK and CD3+ T-cells, while a significant increase in PMN-MDSCs (CD11b^+^HLADR^dim^CD33^+^CD14^−^) was observed. Furthermore, PMN-MDSCs were associated with superior disease-free survival (DFS) in cHL patients. Notably, M-MDSCs (CD11b^+^HLADR^−^CD33^+^CD14^+^) did not differ significantly compared to healthy donors highlighting that several parameters, including patient cohorts, disease stage, immunophenotypic criteria of MDSCs, may influence the different results obtained in the aforementioned studies. 

### 8.3. B-Cell Chronic Lymphocytic Leukemia (B-CLL)

B-CLL represents the most common leukemia in Western countries. The microenvironment appears to play an important role in the pathogenesis of CLL, while immune dysregulation is an early characteristic of the disease, which manifests with hypoglobulinemia, autoimmune phenomena as well as qualitative and quantitative disturbances of T- and NK-cells. MDSCs have also been studied in CLL cases, and the main findings are reviewed below. 

In an initial report, the authors assessed the phenotype of CD14^+^ monocytes in the blood of 29 CLL patients and 15 healthy volunteers [102]. They revealed that CD14^+^ monocytes in CLL patients exhibited reduced staining for HLA-DR than age-matched healthy volunteers and expressed reduced levels of CD86, indicative of decreased antigen-presenting ability with reduced immune stimulatory capacity, thus resembling the phenotype of M-MDSCs. Furthermore, in patients who were in sustained remission at 12 months after completion of therapy (*n* = 18), the median frequency of CD14^+^HLA-DR^lo/neg^ monocytes decreased to a value similar to healthy volunteers. The six patients with higher CD14^+^HLADR^lo/neg^ monocytes at diagnosis (≥2.5 standard deviations above the healthy volunteer mean) had a shorter time to disease progression (median 6.9 months) compared to 19.1 months for patients with lower levels. All this data supports the notion that MDSCs may represent a biomarker of increased tumor burden and poor prognosis in CLL and an important player in its pathogenesis.

Next, Jitschin et al. provided further evidence for a significant increase in M-MDSC frequencies in CLL patients compared to healthy controls [79]. Of importance, by performing an extensive immunostaining, they reported that M-MDSCs express myeloid markers (CD11c, CD13, CD33), adhesion molecules (CD11b, CD62L), receptors associated with promotion, and activation of myeloid cells (TNF-receptor type 2/CD120b), CD115 and CD124 (associated with MDSC activity), and two receptors associated with T-cell anergy and Treg cell induction, such as PDL-1 and CD40. M-MDSCs lack the expression of CD86, CD80, granulocytic markers (CD15, CD66b), as well as CD16 and CD123. By co-culturing monocytes with CLL cells, they showed that the latter induce CD14+HLA-DR^lo^ MDSCs in vitro with T-cell suppressive and Treg cell-promoting capabilities. In addition, gene expression of MDSC-related molecules in healthy monocytes exposed to CLL cells in vitro showed a significant induction of IDO expression, which also involved in the MDSC-mediated suppression. 

In accordance with the above studies, Liu and colleagues reported on the prognostic impact of M-MDSCs in a series of 49 untreated CLL cases, which were compared with 23 CLL-like monoclonal B-cell lymphocytosis (MBL) cases and 21 healthy controls [103]. They showed that M-MDSCs were significantly elevated in CLL patients compared with MBL and control cases and that they were associated with adverse prognostic factors, such as advanced disease stage, CD38 and ZAP-70 expression, as well as IGHV unmutated CLL cases. Finally, they demonstrated that M-MDSCs exert immunosuppressive functions on CD4+ T-cell responses, and the presence of M-MDSCs was inversely correlated with survival. 

Finally, in a study enrolling 50 newly diagnosed CLL patients and 20 age-matched healthy controls, peripheral blood M-MDSCs were found to be increased compared to healthy controls and their presence correlated with the stage of disease (III, VI vs. I) and CD38 and ZAP-70 expression [104]. No significant difference was observed in disease-free survival (DFS) between CLL patients with low M-MDSCs levels (<25% of total monocytes) and high M-MDSCs levels (>25%). However, M-MDSCs levels were associated with overall survival since patients with low M-MDSCs levels had a mean survival time of 43.24 ± 5.02 months compared to 20.6 ± 1.95 months for patients with high M-MDSCs levels. 

### 8.4. MDSCs in T/NK Lymphomas

T-cell lymphomas constitute less than 15% of all NHLs and comprise tumors of mature (post-thymic or peripheral) T-cell origin. They are collectively referred to as peripheral T-cell lymphomas and separated into extranodal, nodal and leukemic forms. With the exception of ALK+ anaplastic T-cell lymphomas, the other T-lymphoma categories usually are associated with poor prognosis. On the other hand, natural killer NK/T lymphomas represent a rare category of lymphomas closely associated with EBV infection and usually presented as an extranodal disease mainly in the nasal/paranasal area, skin and gastrointestinal tract. The majority of NK-T lymphomas have an aggressive clinical course with short survival.

The role of MDSCs has also emerged in these types of lymphomas, where initially, increased levels of PMN-MDSCs were identified in the duodenum of patients with enteropathy-associated T-cell lymphoma (EATL) and associated with the development of EATL through suppression of anti-tumor T-cell immunity [106]. In cutaneous and peripheral T-cell lymphomas, higher levels of M-MDSCs (CD14^+^HLADR^low^) have been observed in the study by Wilcox and colleagues, in which M-MDSCs expressed PDL-1, inhibited T-cell proliferation, and promoted the induction of Foxp3+ Treg cells [105]. Finally, analysis of 32 extranodal NK-lymphoma (ENKL) patients found increased levels of circulating MDSCs compared to 32 healthy controls [59]. MDSCs predominantly consisted of CD14^+^ M-MDSCs (>60%), while PMN-MDSCs comprised approximately 20%. ENKL-MDSCs displayed significantly higher levels of Arg-1, iNOS, and IL-17 and moderate levels of IL-10 and TGFβ compared to healthy donors. In addition, MDSCs suppressed CD4^+^ T-cells via Arg-1, i-NOS, and IL-17. Interestingly, although no correlation was found between MDSCs levels and disease stage, MDSCs levels were independent predictors for DFS and OS. 

## 9. Challenges and Open Questions

The accumulated evidence supports that MDSCs play an important role in the regulation of immune responses in almost all pathologic situations and, therefore, have emerged as potential therapeutic targets. Although, over the last decade, several attempts have been made for the precise characterization of the phenotype and mechanisms of function of MDSCs, the increased heterogeneity of MDSC populations, their plasticity, and also the mosaic inflammatory contexture of the diverse disease pathologies have still hampered the establishment of a unanimous system for MDSC characterization. Furthermore, in the field of malignancies, where MDSCs dominate, most of the knowledge has been derived for solid tumors either in humans or mouse models, while MDSC studies in hematologic malignancies remain limited. Considering that MDSCs are bone marrow-derived cells and most of the hematologic cancers originate in the BM, it is likely that the former may orchestrate the outcome of the latter. It is, therefore, of urgent need to better characterize the MDSC function in hematologic cancers and to delineate how current therapeutic regimens imprint on MDSC to mediate remission or whether MDSCs are involved in therapeutic resistance. Of importance, the assessment of MDSC phenotype and function in BM aspirates is totally ignored and should provide novel insights into the regulation of anti-tumor responses in blood cancers. Towards this, a comprehensive phenotypic characterization of M-MDSCs and PMN-MDSCs should be established across hematologic malignancies since this will allow us to address the aforementioned caveats. Furthermore, the molecular mechanisms via which MDSCs suppress the anti-tumor immunity in hematologic malignancies remain to be determined. Multi-omic analysis of each MDSC subset, such as transcriptomic, proteomic, and genomic data, may shed light on those mechanisms, regarding the functional importance but also recruitment to the periphery (chemokine receptors expressed in MDSC subsets), possible expansion in the BM, differentiation arrest (transcription factors and their post-translational regulation), as well as molecules-proteins that may emerge in each malignant disease setting. In accordance, single-cell RNAseq of total MDSCs may reveal additional MDSC subsets or diverse activation stages, which will facilitate a better categorization of these diseases. Another crucial aspect, which seems to influence the MDSC properties is metabolism, which, in the field of hematology, is largely ignored. Therefore, the determination of metabolites through metabolomic analysis and the assessment of metabolic parameters (such as oxygen consumption rate and extracellular acidification rate) will provide novel insights into the metabolic adaptations of MDSCs in such diseases and will open new avenues for investigations focusing on the metabolic rewiring and therapeutic targeting of MDSCs. 

Interestingly, MDSCs abundantly express the checkpoint molecule PDL1; however, its role in MDSC-mediated suppression remain ill-defined. Considering the promising results obtained with the anti-PD1 (the ligand of PD-L1) checkpoint inhibitor immunotherapy in the treatment of Hodgkin’s lymphoma, it is of great interest to investigate how this therapeutic regimen modulates the function and frequencies of MDSCs. It is almost certain that anti-PD1 immunotherapy targets all PDL1-expressing cells, including MDSCs, and thus, we formulate the hypothesis that such interaction may result in reprogramming this potent suppressive population, thus contributing to therapeutic efficacy. Future studies on MDSCs from anti-PD1-treated patients responding or not responding to therapy may address this hypothesis and provide further evidence on checkpoint inhibitors immunotherapy resistance. In line with this, low frequencies of M-MDSCs were associated with a better response in lymphoma patients treated with the third generation of CD19 Chimeric Antigen Receptor (CAR) T-cells [107]. Considering the powerful therapeutic potential that chimeric antigen receptor (CAR) T-cells hold for patients with lymphomas, but also that MDSCs have been shown to have a detrimental effect on CAR-T-cell expansion and cytolytic function, it is necessary to identify how the expansion of MDSCs, which has been reported in almost all lymphoma studies as discussed above, may interfere with the effectiveness of CAR-T protocols in order to condition the patient accordingly aiming to maximize the therapeutic benefit. In particular, combination of CAR T-cell therapies with immune checkpoint inhibitors, mainly anti-PD1 and anti-PDL1, may significantly increase the anti-tumor immunity in lymphomas, paralleling the suggestions and observations in solid tumors [108]. In contrast, MDSCs have been emerged as a potential therapeutic approach in graft-versus-host disease (GVHD), which constitute a major impediment of the allogeneic stem cell transplantation (allo-HSCT). Briefly, allo-HSCT is a curative treatment for hematologic malignancies; however, its efficacy is compromised by the development of GvHD, while immunosuppressive treatment which ameliorate GvHD associate with malignancy recurrence since also affect the graft-versus-leukemia effect (GVL). In this regard, MDSCs have been shown to inhibit GVHD without affecting GVL mainly in mouse transplantation settings [109], while in humans, this field remains in its infancy and should be readily explored.

## 10. Conclusions

In lymphomas, which constitute malignancies of the immune system, immunosuppressive cells orchestrate the anti-tumor immunity, which is further strengthened by the therapeutic efficacy of immune checkpoint inhibitors in lymphoma patients. Therefore, unravelling the phenotype and functional cues of immunosuppressive cells, such as MDSCs, will provide novel insights not only in the pathogenetic mechanisms of such complex and heterogenous malignancies but also set the stage for the identification of biomarkers for the prediction of the response to various treatments. Finally, the delineation of the suppressive programs via which MDSCs modulate the immune responses in individuals with lymphoma may provide novel therapeutic targets to ensure long-lasting remission with diminished toxicities.

## Figures and Tables

**Table 1:** MDSC subsets in different types of lymphomas.

Lymphoma Type	MDSCs Characterization	Mode of Action/Expansion	Clinical Implications	Reference
DLBCL Ν = 23 ND	CD14^+^HLADR^low/−^ (M-MDSCs)	-	Association with disease activity	2011 [60]
DLBCL N = 144 ND	CD14^+^HLADR^low/−^ (M-MDSCs)	-	Significant difference of M-MDSC between GCB-DLBCL and non-GCB-DLBCL patients	2015 [95] 2016 [96]
DLBCL N = 66 ND	Lin^neg^HLA-DR^low/−^ CD123^−^CD33^+^CD11b^+^ (PMN-MDSCs) CD3^−^CD335^−^CD115^+^ CD14^+^ HLA-DR ^low^ (M-MDSCs)	IL-10, S100A12 and programmed death-ligand 1 (PD-L1)	Increased PMN- and M-MDSC compared to HC; Only M-MDSCs corelated with high-risk features and poor outcome	2016 [97]
DLBCL TOTAL = 103 N = 65 ND 12 RELAPSED 26 REMISSION	CD14^+^HLA-DR^low/−^ (M-MDSCs)	IL-35	MDSCs levels correlated with disease progression	2021 [94]
NHLs, N = 40 R/R = 36, ND = 4 DLBCL = 18 FL = 14, MCL = 4, SLL = 2,MALT = 1, LPL = 1	CD14^+^HLA-DR^low/−^ CD120b^low^ (M-MDSCs)	Arginine metabolism.	increased M-MDSCs correlated with more aggressive disease	2011 [61]
NHLs, N = 42 R/R = 26, ND = 16 DLBCL = 21, FL = 16, NHLi = 5	CD14^+^HLA-DR^low/−^ (M-MDSCs)	arginase I	Increased in NHL vs. HC Correlated with: Advanced stage of disease (III/IV), aggressive histology and relapsed/refractory disease.	2014 [62]
NHL, N = 22 (ND) DLBCL = 6, FL = 7, MCL = 4, MZL = 5	CD14^+^HLA-DR^low/−^ (M-MDSCs)	IL-10	elevated in NHL vs. HC Correlated with higher IPI.	2015 [98]
HODGKIN ND N = 60	CD14^+^HLA-DR^low/−^ (M-MDSCs) CD11b^+^CD33^+^CD14^−^HLA-DR^−^ Lin^−^ (PMN-MDSCs) CD11b^+^CD33^+^CD14^–^HLA-DR^−^CD34^+^ (CD34^+^MDSCs)	-	All MDSCs were increased in HL vs. HC CD34^+^MDSCs were strong predictors for PFS	2015 [99]
NHL+HL, ND N = 124 HL = 31, DLBCL = 62 NHLi = 31	CD66b^+^CD33^dim^HLADR^−^ (PMN-MDSCs)		PMN-MDSCs were increased in all NHL+HL vs. HC	2016 [100]
NHL+HL, N = 43 ND DLBCL = 24 HL = 19	CD11b^+^HLA-DR^dim^CD33^dim^ CD14^−^ (PMN-MDSCs) CD11b^+^HLADR^−^CD33^+^CD14^+^ (M-MDSCs)	-	Only PMN-MDSCs were increased in NHL+HL vs. HC Associated with better DFS in CHL	2019 [101]
B-CLL, N = 29 untreated	CD14^+^HLADR^lo/neg^ (M-MDSCs)	Diminished HLA-DR and CD86 expression	MDSCs correlated with disease progression MDSCs decreased to normal at remission	2012 [102]
B-CLL, N = 79 Untreated	CD14^+^HLADR^lo/neg^ (M-MDSCs)	Induction of Tregs and indoleamine 2,3-dioxygenase (IDO) activity	Increased in CLL vs. HC Associated with shorter LDT	2014 [79]
B-CLL, N = 49 MBL, N = 23 Untreated	CD14^+^HLA-DR ^low/–^ (M-MDSCs)	-	Increased in CLL vs. MBL vs. HC MDSCs. Associated with advanced disease stage, IGHV unmutated CLL cases and worse OS	2015 [103]
B-CLL, N = 50	CD14^+^HLA-DR ^low/–^ (M-MDSCs)	-	Increased in CLL vs. HC correlated with disease stage, disease progression and OS	2020 [104]
Cutaneous and Peripheral T-cell lymphomas	CD14^+^HLA-DR ^low/–^ (M-MDSCs) PBMCs	-	M-MDSCs expressing PD-L1 highly expand in CTCL patients	2009 [105]
Extranodal NK, N = 32	CD14^+^HLADR^−^CD33^+^CD11b^+^ (MDSCs) HLADR^−^CD33^+^CD11b^+^ CD15^+^ (PMN-MDSCs)	Arg-1, i-NOS and IL-17	Higher levels in NHL vs. HC; M-MDSCs were independent predictors for DFS and OS.	2015 [59]

Abbreviations: NHL: Non Hodgkin Lymphoma; HC: Healthy Controls; DFS: Disease Free Survival; OS: Overall Survival; NK: Natural Killer; B-CLL: B-Chronic Lymphocytic Leukemia; CTCL: Cutaneous T-cell Lymphoma; ND: Newly Diagnosed; DLBCL: Diffuse Large B-cell Lymphoma; FL: Follicular Lymphoma; MCL: Mantle Cell Lymphoma; HL: Hodgkin Lymphoma; MBL: Monoclonal B-cell Lymphocytosis; SLL: Small Lymphocytic Lymphoma; MALT: Mucosa- associated Lymphoid Tissue; LPL: Lymphoplasmacytic Lymphoma; GCB: Germinal Center B-cell; R/R: Relapsed/Refractory.

## References

[1] Bennett J.A., Srinivasa Rao V., Mitchell M.S. (1978). Systemic Bacillus Calmette-Guerin (BCG) Activates Natural Suppressor Cells. Proc. Natl. Acad. Sci. USA.

[2] Talmadge J.E., Gabrilovich D.I. (2013). History of Myeloid-Derived Suppressor Cells. Nat. Rev. Cancer.

[3] Gabrilovich D.I., Bronte V., Chen S.H., Colombo M.P., Ochoa A., Ostrand-Rosenberg S., Schreiber H. (2007). The Terminology Issue for Myeloid-Derived Suppressor Cells. Cancer Res..

[4] Yang R., Roden R.B. (2007). The Terminology Issue for Myeloid-Derived Suppressor Cells. Cancer Res..

[5] Veglia F., Sanseviero E., Gabrilovich D.I. (2021). Myeloid-Derived Suppressor Cells in the Era of Increasing Myeloid Cell Diversity. Nat. Rev. Immunol..

[6] Gabrilovich D.I., Ostrand-Rosenberg S., Bronte V. (2012). Coordinated Regulation of Myeloid Cells by Tumours. Nat. Rev. Immunol..

[7] Ioannou M., Alissafi T., Lazaridis I., Deraos G., Matsoukas J., Gravanis A., Mastorodemos V., Plaitakis A., Sharpe A., Boumpas D. (2012). Crucial Role of Granulocytic Myeloid-Derived Suppressor Cells in the Regulation of Central Nervous System Autoimmune Disease. J. Immunol..

[8] Ioannou M., Alissafi T., Boon L., Boumpas D., Verginis P. (2013). In Vivo Ablation of Plasmacytoid Dendritic Cells Inhibits Autoimmunity through Expansion of Myeloid-Derived Suppressor Cells. J. Immunol..

[9] Vlachou K., Mintzas K., Glymenaki M., Ioannou M., Papadaki G., Bertsias G.K., Sidiropoulos P., Boumpas D.T., Verginis P. (2016). Elimination of Granulocytic Myeloid-Derived Suppressor Cells in Lupus-Prone Mice Linked to Reactive Oxygen Species-Dependent Extracellular Trap Formation. Arthritis Rheumatol..

[10] Wood K.J., Bushell A., Hester J. (2012). Regulatory Immune Cells in Transplantation. Nat. Rev. Immunol..

[11] Bronte V., Brandau S., Chen S.H., Colombo M.P., Frey A.B., Greten T.F., Mandruzzato S., Murray P.J., Ochoa A., Ostrand-Rosenberg S. (2016). Recommendations for Myeloid-Derived Suppressor Cell Nomenclature and Characterization Standards. Nat. Commun..

[12] Kusmartsev S.A., Li Y., Chen S.H. (2000). Gr-1+ Myeloid Cells Derived from Tumor-Bearing Mice Inhibit Primary T Cell Activation Induced through CD3/CD28 Costimulation. J. Immunol..

[13] Gabrilovich D., Ishida T., Oyama T., Ran S., Kravtsov V., Nadaf S., Carbone D.P. (1998). Vascular Endothelial Growth Factor Inhibits the Development of Dendritic Cells and Dramatically Affects the Differentiation of Multiple Hematopoietic Lineages in Vivo. Blood.

[14] Watson G.A., Fu Y.X., Lopez D.M. (1991). Splenic Macrophages from Tumor-Bearing Mice Co-Expressing MAC-1 and MAC-2 Antigens Exert Immunoregulatory Functions via Two Distinct Mechanisms. J. Leukoc. Biol..

[15] Youn J.I., Nagaraj S., Collazo M., Gabrilovich D.I. (2008). Subsets of Myeloid-Derived Suppressor Cells in Tumor-Bearing Mice. J. Immunol..

[16] Dumitru C.A., Moses K., Trellakis S., Lang S., Brandau S. (2012). Neutrophils and Granulocytic Myeloid-Derived Suppressor Cells: Immunophenotyping, Cell Biology and Clinical Relevance in Human Oncology. Cancer Immunol. Immunother..

[17] Mandruzzato S., Brandau S., Britten C.M., Bronte V., Damuzzo V., Gouttefangeas C., Maurer D., Ottensmeier C., Van der Burg S.H., Welters M.J. (2016). Toward Harmonized Phenotyping of Human Myeloid-Derived Suppressor Cells by Flow Cytometry: Results from an Interim Study. Cancer Immunol. Immunother..

[18] Condamine T., Dominguez G.A., Youn J.I., Kossenkov A.V., Mony S., Alicea-Torres K., Tcyganov E., Hashimoto A., Nefedova Y., Lin C. (2016). Lectin-Type Oxidized LDL Receptor-1 Distinguishes Population of Human Polymorphonuclear Myeloid-Derived Suppressor Cells in Cancer Patients. Sci. Immunol..

[19] Nan J., Xing Y.F., Hu B., Tang J.X., Dong H.M., He Y.M., Ruan D.Y., Ye Q.J., Cai J.R., Ma X.K. (2018). Endoplasmic Reticulum Stress Induced LOX-1+CD15+ Polymorphonuclear Myeloid-Derived Suppressor Cells in Hepatocellular Carcinoma. Immunology.

[20] Kim H.R., Park S.M., Seo S.U., Jung I., Yoon H.I., Gabrilovich D.I., Cho B.C., Seong S.Y., Ha S.J., Youn J.I. (2018). The Ratio of Peripheral Regulatory T Cells to Lox-1+ Polymorphonuclear Myeloid-Derived Suppressor Cells Predicts the Early Response to Anti–PD-1 Therapy in Patients with Non–Small Cell Lung Cancer. Am. J. Respir. Crit. Care Med..

[21] Chai E., Zhang L., Li C. (2019). LOX-1+ PMN-MDSC Enhances Immune Suppression Which Promotes Glioblastoma Multiforme Progression. Cancer Manag. Res..

[22] Si Y., Merz S.F., Jansen P., Wang B., Bruderek K., Altenhoff P., Mattheis S., Lang S., Gunzer M., Klode J. (2019). Multidimensional Imaging Provides Evidence for Down-Regulation of T Cell Effector Function by MDSC in Human Cancer Tissue. Sci. Immunol..

[23] Tavukcuoglu E., Horzum U., Yanik H., Uner A., Yoyen-Ermis D., Nural S.K., Aydin B., Sokmensuer C., Karakoc D., Yilmaz K.B. (2020). Human Splenic Polymorphonuclear Myeloid-Derived Suppressor Cells (PMN-MDSC) Are Strategically Located Immune Regulatory Cells in Cancer. Eur. J. Immunol..

[24] Bronte V., Chappell D.B., Apolloni E., Cabrelle A., Wang M., Hwu P., Restifo N.P. (1999). Unopposed Production of Granulocyte-Macrophage Colony-Stimulating Factor by Tumors Inhibits CD8+ T Cell Responses by Dysregulating Antigen-Presenting Cell Maturation. J. Immunol..

[25] Waight J.D., Hu Q., Miller A., Liu S., Abrams S.I. (2011). Tumor-Derived G-CSF Facilitates Neoplastic Growth through a Granulocytic Myeloid-Derived Suppressor Cell-Dependent Mechanism. PLoS ONE.

[26] Sawanobori Y., Ueha S., Kurachi M., Shimaoka T., Talmadge J.E., Abe J., Shono Y., Kitabatake M., Kakimi K., Mukaida N. (2008). Chemokine-Mediated Rapid Turnover of Myeloid-Derived Suppressor Cells in Tumor-Bearing Mice. Blood.

[27] Pan P.Y., Wang G.X., Yin B., Ozao J., Ku T., Divino C.M., Chen S.H. (2008). Reversion of Immune Tolerance in Advanced Malignancy: Modulation of Myeloid-Derived Suppressor Cell Development by Blockade of Stem-Cell Factor Function. Blood.

[28] Bunt S.K., Yang L., Sinha P., Clements V.K., Leips J., Ostrand-Rosenberg S. (2007). Reduced Inflammation in the Tumor Microenvironment Delays the Accumulation of Myeloid-Derived Suppressor Cells and Limits Tumor Progression. Cancer Res..

[29] Sinha P., Okoro C., Foell D., Freeze H.H., Ostrand-Rosenberg S., Srikrishna G. (2008). Proinflammatory S100 Proteins Regulate the Accumulation of Myeloid-Derived Suppressor Cells. J. Immunol..

[30] Sinha P., Clements V.K., Fulton A.M., Ostrand-Rosenberg S. (2007). Prostaglandin E2 Promotes Tumor Progression by Inducing Myeloid-Derived Suppressor Cells. Cancer Res..

[31] Ochoa A.C., Zea A.H., Hernandez C., Rodriguez P.C. (2007). Arginase, Prostaglandins, and Myeloid-Derived Suppressor Cells in Renal Cell Carcinoma. Clin. Cancer Res..

[32] Condamine T., Gabrilovich D.I. (2011). Molecular Mechanisms Regulating Myeloid-Derived Suppressor Cell Differentiation and Function. Trends Immunol..

[33] Condamine T., Mastio J., Gabrilovich D.I. (2015). Transcriptional Regulation of Myeloid-Derived Suppressor Cells. J. Leukoc. Biol..

[34] Wang W., Xia X., Mao L., Wang S. (2019). The CCAAT/Enhancer-Binding Protein Family: Its Roles in MDSC Expansion and Function. Front. Immunol..

[35] Gabrilovich D.I., Nagaraj S. (2009). Myeloid-Derived Suppressor Cells as Regulators of the Immune System. Nat. Rev. Immunol..

[36] Hatziioannou A., Alissafi T., Verginis P. (2017). Myeloid-Derived Suppressor Cells and T Regulatory Cells in Tumors: Unraveling the Dark Side of the Force. J. Leukoc. Biol..

[37] Millrud C.R., Bergenfelz C., Leandersson K. (2017). On the Origin of Myeloid-Derived Suppressor Cells. Oncotarget.

[38] Murdoch C., Giannoudis A., Lewis C.E. (2004). Mechanisms Regulating the Recruitment of Macrophages into Hypoxic Areas of Tumors and Other Ischemic Tissues. Blood.

[39] Chun E., Lavoie S., Michaud M., Gallini C.A., Kim J., Soucy G., Odze R., Glickman J.N., Garrett W.S. (2015). CCL2 Promotes Colorectal Carcinogenesis by Enhancing Polymorphonuclear Myeloid-Derived Suppressor Cell Population and Function. Cell Rep..

[40] Huang B., Lei Z., Zhao J., Gong W., Liu J., Chen Z., Liu Y., Li D., Yuan Y., Zhang G.M. (2007). CCL2/CCR2 Pathway Mediates Recruitment of Myeloid Suppressor Cells to Cancers. Cancer Lett..

[41] Chang A.L., Miska J., Wainwright D.A., Dey M., Rivetta C.V., Yu D., Kanojia D., Pituch K.C., Qiao J., Pytel P. (2016). CCL2 Produced by the Glioma Microenvironment Is Essential for the Recruitment of Regulatory T Cells and Myeloid-Derived Suppressor Cells. Cancer Res..

[42] Luboshits G., Shina S., Kaplan O., Engelberg S., Nass D., Lifshitz-Mercer B., Chaitchik S., Keydar I., Ben-Baruch A. (1999). Elevated Expression of the CC Chemokine Regulated on Activation, Normal T Cell Expressed and Secreted (RANTES) in Advanced Breast Carcinoma. Cancer Res..

[43] Zhang Y., Lv D., Kim H.J., Kurt R.A., Bu W., Li Y., Ma X. (2013). A Novel Role of Hematopoietic CCL5 in Promoting Triple-Negative Mammary Tumor Progression by Regulating Generation of Myeloid-Derived Suppressor Cells. Cell Res..

[44] Ichikawa M., Williams R., Wang L., Vogl T., Srikrishna G. (2011). S100A8/A9 Activate Key Genes and Pathways in Colon Tumor Progression. Mol. Cancer Res..

[45] Inamoto S., Itatani Y., Yamamoto T., Minamiguchi S., Hirai H., Iwamoto M., Hasegawa S., Taketo M.M., Sakai Y., Kawada K. (2016). Loss of SMAD4 Promotes Colorectal Cancer Progression by Accumulation of Myeloid-Derived Suppressor Cells through the CCL15–CCR1 Chemokine Axis. Clin. Cancer Res..

[46] Chiu D.K., Xu I.M., Lai R.K., Tse A.P., Wei L.L., Koh H.Y., Li L.L., Lee D., Lo R.C., Wong C.M. (2016). Hypoxia Induces Myeloid-Derived Suppressor Cell Recruitment to Hepatocellular Carcinoma through Chemokine (C-C Motif) Ligand 26. Hepatology.

[47] Alfaro C., Teijeira A., Oñate C., Pérez G., Sanmamed M.F., Andueza M.P., Alignani D., Labiano S., Azpilikueta A., Rodriguez-Paulete A. (2016). Tumor-Produced Interleukin-8 Attracts Human Myeloid-Derived Suppressor Cells and Elicits Extrusion of Neutrophil Extracellular Traps (NETs). Clin. Cancer Res..

[48] Obermajer N., Muthuswamy R., Odunsi K., Edwards R.P., Kalinski P. (2011). PGE(2)-Induced CXCL12 Production and CXCR4 Expression Controls the Accumulation of Human MDSCs in Ovarian Cancer Environment. Cancer Res..

[49] Dolcetti L., Peranzoni E., Ugel S., Marigo I., Fernandez Gomez A., Mesa C., Geilich M., Winkels G., Traggiai E., Casati A. (2010). Hierarchy of Immunosuppressive Strength among Myeloid-Derived Suppressor Cell Subsets Is Determined by GM-CSF. Eur. J. Immunol..

[50] Movahedi K., Guilliams M., Van den Bossche J., Van den Bergh R., Gysemans C., Beschin A., De Baetselier P., Van Ginderachter J.A. (2008). Identification of Discrete Tumor-Induced Myeloid-Derived Suppressor Cell Subpopulations with Distinct T Cell–Suppressive Activity. Blood.

[51] Bronte V., Zanovello P. (2005). Regulation of Immune Responses by L-Arginine Metabolism. Nat. Rev. Immunol..

[52] Rodríguez P.C., Ochoa A.C. (2008). Arginine Regulation by Myeloid Derived Suppressor Cells and Tolerance in Cancer: Mechanisms and Therapeutic Perspectives. Immunol. Rev..

[53] Rodriguez P.C., Zea A.H., Culotta K.S., Zabaleta J., Ochoa J.B., Ochoa A.C. (2002). Regulation of t cell receptor cd3ζ chain expression byl-arginine. J. Biol. Chem..

[54] Rodriguez P.C., Quiceno D.G., Ochoa A.C. (2007). L-Arginine Availability Regulates T-Lymphocyte Cell-Cycle Progression. Blood.

[55] Harari O., Liao J.K. (2004). Inhibition of MHC II Gene Transcription by Nitric Oxide and Antioxidants. Curr. Pharm. Des..

[56] Mazzoni A., Bronte V., Visintin A., Spitzer J.H., Apolloni E., Serafini P., Zanovello P., Segal D.M. (2002). Myeloid Suppressor Lines Inhibit T Cell Responses by an NO-Dependent Mechanism. J. Immunol..

[57] Apolloni E., Bronte V., Mazzoni A., Serafini P., Cabrelle A., Segal D.M., Young H.A., Zanovello P. (2000). Immortalized Myeloid Suppressor Cells Trigger Apoptosis in Antigen-Activated T Lymphocytes. J. Immunol..

[58] Koblish H.K., Hunter C.A., Wysocka M., Trinchieri G., Lee W.M. (1998). Immune Suppression by Recombinant Interleukin (RIL)-12 Involves Interferon Gamma Induction of Nitric Oxide Synthase 2 (INOS) Activity: Inhibitors of NO Generation Reveal the Extent of RIL-12 Vaccine Adjuvant Effect. J. Exp. Med..

[59] Zhang H., Li Z.L., Ye S.B., Ouyang L.Y., Chen Y.S., He J., Huang H.Q., Zeng Y.X., Zhang X.S., Li J. (2015). Myeloid-Derived Suppressor Cells Inhibit T Cell Proliferation in Human Extranodal NK/T Cell Lymphoma: A Novel Prognostic Indicator. Cancer Immunol. Immunother..

[60] Tadmor T., Fell R., Polliack A., Attias D. (2013). Absolute Monocytosis at Diagnosis Correlates with Survival in Diffuse Large B-Cell Lymphoma—Possible Link with Monocytic Myeloid-Derived Suppressor Cells. Hematol. Oncol..

[61] Lin Y., Gustafson M.P., Bulur P.A., Gastineau D.A., Witzig T.E., Dietz A.B. (2011). Immunosuppressive CD14+HLA-DR(Low)/-Monocytes in B-Cell Non-Hodgkin Lymphoma. Blood.

[62] Khalifa K.A., Badawy H.M., Radwan W.M., Shehata M.A., Bassuoni M.A. (2014). CD14+ HLA-DR Low/− Monocytes as Indicator of Disease Aggressiveness in B-Cell Non-Hodgkin Lymphoma. Int. J. Lab. Hematol..

[63] Görgün G.T., Whitehill G., Anderson J.L., Hideshima T., Maguire C., Laubach J., Raje N., Munshi N.C., Richardson P.G., Anderson K.C. (2013). Tumor-Promoting Immune-Suppressive Myeloid-Derived Suppressor Cells in the Multiple Myeloma Microenvironment in Humans. Blood.

[64] Giallongo C., Tibullo D., Parrinello N.L., La Cava P., Di Rosa M., Bramanti V., Di Raimondo C., Conticello C., Chiarenza A., Palumbo G.A. (2016). Granulocyte-like Myeloid Derived Suppressor Cells (G-MDSC) Are Increased in Multiple Myeloma and Are Driven by Dysfunctional Mesenchymal Stem Cells (MSC). Oncotarget.

[65] Romano A., Parrinello N.L., La Cava P., Tibullo D., Giallongo C., Camiolo G., Puglisi F., Parisi M., Pirosa M.C., Martino E. (2018). PMN-MDSC and Arginase Are Increased in Myeloma and May Contribute to Resistance to Therapy. Expert Rev. Mol. Diagn..

[66] Srivastava M.K., Sinha P., Clements V.K., Rodriguez P., Ostrand-Rosenberg S. (2010). Myeloid-Derived Suppressor Cells Inhibit T-Cell Activation by Depleting Cystine and Cysteine. Cancer Res..

[67] Liu M., Wang X., Wang L., Ma X., Gong Z., Zhang S., Li Y. (2018). Targeting the IDO1 Pathway in Cancer: From Bench to Bedside. J. Hematol. Oncol..

[68] Platten M., Nollen E.A.A., Röhrig U.F., Fallarino F., Opitz C.A. (2019). Tryptophan Metabolism as a Common Therapeutic Target in Cancer, Neurodegeneration and Beyond. Nat. Rev. Drug Discov..

[69] Corzo C.A., Cotter M.J., Cheng P., Cheng F., Kusmartsev S., Sotomayor E., Padhya T., McCaffrey T.V., McCaffrey J.C., Gabrilovich D.I. (2009). Mechanism Regulating Reactive Oxygen Species in Tumor-Induced Myeloid-Derived Suppressor Cells. J. Immunol..

[70] Kusmartsev S., Nefedova Y., Yoder D., Gabrilovich D.I. (2004). Antigen-Specific Inhibition of CD8+ T Cell Response by Immature Myeloid Cells in Cancer Is Mediated by Reactive Oxygen Species. J. Immunol..

[71] Schmielau J., Finn O.J. (2001). Activated Granulocytes and Granulocyte-Derived Hydrogen Peroxide Are the Underlying Mechanism of Suppression of T-Cell Function in Advanced Cancer Patients. Cancer Res..

[72] Szuster-Ciesielska A., Hryciuk-Umer E., Stepulak A., Kupisz K., Kandefer-Szerszeń M. (2004). Reactive Oxygen Species Production by Blood Neutrophils of Patients with Laryngeal Carcinoma and Antioxidative Enzyme Activity in Their Blood. Acta Oncol..

[73] Geskin L.J., Akilov O.E., Kwon S., Schowalter M., Watkins S., Whiteside T.L., Butterfield L.H., Falo L.D. (2018). Therapeutic Reduction of Cell-Mediated Immunosuppression in Mycosis Fungoides and Sézary Syndrome. Cancer Immunol. Immunother..

[74] Ohl K., Tenbrock K. (2018). Reactive Oxygen Species as Regulators of MDSC-Mediated Immune Suppression. Front. Immunol..

[75] Ku A.W., Muhitch J.B., Powers C.A., Diehl M., Kim M., Fisher D.T., Sharda A.P., Clements V.K., O’Loughlin K., Minderman H. (2016). Tumor-Induced MDSC Act via Remote Control to Inhibit L-Selectin-Dependent Adaptive Immunity in Lymph Nodes. Elife.

[76] Ostrand-Rosenberg S., Sinha P. (2009). Myeloid-Derived Suppressor Cells: Linking Inflammation and Cancer. J. Immunol..

[77] Hanson E.M., Clements V.K., Sinha P., Ilkovitch D., Ostrand-Rosenberg S. (2009). Myeloid-Derived Suppressor Cells down-Regulate L-Selectin Expression on CD4+ and CD8+ T Cells. J. Immunol..

[78] Huang B., Pan P.Y., Li Q., Sato A.I., Levy D.E., Bromberg J., Divino C.M., Chen S.H. (2006). Gr-1+CD115+ Immature Myeloid Suppressor Cells Mediate the Development of Tumor-Induced T Regulatory Cells and T-Cell Anergy in Tumor-Bearing Host. Cancer Res..

[79] Jitschin R., Braun M., Büttner M., Dettmer-Wilde K., Bricks J., Berger J., Eckart M.J., Krause S.W., Oefner P.J., Le Blanc K. (2014). CLL-Cells Induce IDOhi CD14+HLA-DRlo Myeloid-Derived Suppressor Cells That Inhibit T-Cell Responses and Promote TRegs. Blood.

[80] Beury D.W., Parker K.H., Nyandjo M., Sinha P., Carter K.A., Ostrand-Rosenberg S. (2014). Cross-Talk among Myeloid-Derived Suppressor Cells, Macrophages, and Tumor Cells Impacts the Inflammatory Milieu of Solid Tumors. J. Leukoc. Biol..

[81] Li H., Han Y., Guo Q., Zhang M., Cao X. (2009). Cancer-Expanded Myeloid-Derived Suppressor Cells Induce Anergy of NK Cells through Membrane-Bound TGF-Β1. J. Immunol..

[82] Alissafi T., Hatzioannou A., Mintzas K., Barouni R.M., Banos A., Sormendi S., Polyzos A., Xilouri M., Wielockx B., Gogas H. (2018). Autophagy Orchestrates the Regulatory Program of Tumor-Associated Myeloid-Derived Suppressor Cells. J. Clin. Investig..

[83] Kalafati L., Kourtzelis I., Schulte-Schrepping J., Li X., Hatzioannou A., Grinenko T., Hagag E., Sinha A., Has C., Dietz S. (2020). Innate Immune Training of Granulopoiesis Promotes Anti-Tumor Activity. Cell.

[84] Bizymi N., Bjelica S., Kittang A.O., Mojsilovic S., Velegraki M., Pontikoglou C., Roussel M., Ersvær E., Santibañez J.F., Lipoldová M. (2019). Myeloid-Derived Suppressor Cells in Hematologic Diseases: Promising Biomarkers and Treatment Targets. HemaSphere.

[85] Serafini P., Mgebroff S., Noonan K., Borrello I. (2008). Myeloid-Derived Suppressor Cells Promote Cross-Tolerance in B-Cell Lymphoma by Expanding Regulatory T Cells. Cancer Res..

[86] Xu Z., Ji J., Xu J., Li D., Shi G., Liu F., Ding L., Ren J., Dou H., Wang T. (2017). MiR-30a Increases MDSC Differentiation and Immunosuppressive Function by Targeting SOCS3 in Mice with B-Cell Lymphoma. FEBS J..

[87] Han S., Jeong A.L., Lee S., Park J.S., Kim K.D., Choi I., Yoon S.R., Lee M.S., Lim J.S., Han S.H. (2013). Adiponectin Deficiency Suppresses Lymphoma Growth in Mice by Modulating NK Cells, CD8 T Cells, and Myeloid-Derived Suppressor Cells. J. Immunol..

[88] Abedi-Valugerdi M., Wolfsberger J., Pillai P.R., Zheng W., Sadeghi B., Zhao Y., Hassan M. (2016). Suppressive Effects of Low-Dose 5-Fluorouracil, Busulfan or Treosulfan on the Expansion of Circulatory Neutrophils and Myeloid Derived Immunosuppressor Cells in Tumor-Bearing Mice. Int. Immunopharmacol..

[89] Abedi-Valugerdi M., Zheng W., Benkessou F., Zhao Y., Hassan M. (2017). Differential Effects of Low-Dose Fludarabine or 5-Fluorouracil on the Tumor Growth and Myeloid Derived Immunosuppression Status of Tumor-Bearing Mice. Int. Immunopharmacol..

[90] Pilot T., Fratti A., Thinselin C., Perrichet A., Demontoux L., Limagne E., Derangère V., Ilie A., Ndiaye M., Jacquin E. (2020). Heat Shock and HSP70 Regulate 5-FU-Mediated Caspase-1 Activation in Myeloid-Derived Suppressor Cells and Tumor Growth in Mice. J. Immunother. Cancer.

[91] Qin H., Lerman B., Sakamaki I., Wei G., Cha S.C., Rao S.S., Qian J., Hailemichael Y., Nurieva R., Dwyer K.C. (2014). Generation of a New Therapeutic Peptide That Depletes Myeloid-Derived Suppressor Cells in Tumor-Bearing Mice. Nat. Med..

[92] Sakamaki I., Kwak L.W., Cha S.C., Yi Q., Lerman B., Chen J., Surapaneni S., Bateman S., Qin H. (2014). Lenalidomide Enhances the Protective Effect of a Therapeutic Vaccine and Reverses Immune Suppression in Mice Bearing Established Lymphomas. Leukemia.

[93] Lu F., Zhao Y., Pang Y., Ji M., Sun Y., Wang H., Zou J., Wang Y., Li G., Sun T. (2021). NLRP3 Inflammasome Upregulates PD-L1 Expression and Contributes to Immune Suppression in Lymphoma. Cancer Lett..

[94] Wang Z., Jiang R., Li Q., Wang H., Tao Q., Zhai Z. (2021). Elevated M-MDSCs in Circulation Are Indicative of Poor Prognosis in Diffuse Large B-Cell Lymphoma Patients. J. Clin. Med..

[95] Wu C., Wu X., Zhang X., Chai Y., Guo Q., Li L., Yue L., Bai J., Wang Z., Zhang L. (2015). Prognostic Significance of Peripheral Monocytic Myeloid-Derived Suppressor Cells and Monocytes in Patients Newly Diagnosed with Diffuse Large b-Cell Lymphoma. Int. J. Clin. Exp. Med..

[96] Wu C., Wu X., Liu X., Yang P., Xu J., Chai Y., Guo Q., Wang Z., Zhang L. (2016). Prognostic Significance of Monocytes and Monocytic Myeloid-Derived Suppressor Cells in Diffuse Large B-Cell Lymphoma Treated with R-CHOP. Cell. Physiol. Biochem..

[97] Azzaoui I., Uhel F., Rossille D., Pangault C., Dulong J., Le Priol J., Lamy T., Houot R., Le Gouill S., Cartron G. (2016). T-Cell Defect in Diffuse Large B-Cell Lymphomas Involves Expansion of Myeloid-Derived Suppressor Cells. Blood.

[98] Xiu B., Lin Y., Grote D.M., Ziesmer S.C., Gustafson M.P., Maas M.L., Zhang Z., Dietz A.B., Porrata L.F., Novak A.J. (2015). IL-10 Induces the Development of Immunosuppressive CD14+ HLA-DRlow/- Monocytes in B-Cell Non-Hodgkin Lymphoma. Blood Cancer J..

[99] Romano A., Parrinello N.L., Vetro C., Forte S., Chiarenza A., Figuera A., Motta G., Palumbo G.A., Ippolito M., Consoli U. (2015). Circulating Myeloid-Derived Suppressor Cells Correlate with Clinical Outcome in Hodgkin Lymphoma Patients Treated up-Front with a Risk-Adapted Strategy. Br. J. Haematol..

[100] Marini O., Spina C., Mimiola E., Cassaro A., Malerba G., Todeschini G., Perbellini O., Scupoli M., Carli G., Facchinelli D. (2016). Identification of Granulocytic Myeloid-Derived Suppressor Cells (G-MDSCs) in the Peripheral Blood of Hodgkin and Non-Hodgkin Lymphoma Patients. Oncotarget.

[101] Amini R.M., Enblad G., Hollander P., Laszlo S., Eriksson E., Ayoola Gustafsson K., Loskog A., Thörn I. (2019). Altered Profile of Immune Regulatory Cells in the Peripheral Blood of Lymphoma Patients. BMC Cancer.

[102] Gustafson M.P., Abraham R.S., Lin Y., Wu W., Gastineau D.A., Zent C.S., Dietz A.B. (2012). Association of an Increased Frequency of CD14 +HLA-DR Lo/Neg Monocytes with Decreased Time to Progression in Chronic Lymphocytic Leukaemia (CLL). Br. J. Haematol..

[103] Liu J., Zhou Y., Huang Q., Qiu L. (2015). CD14+HLA-DRlow/- Expression: A Novel Prognostic Factor in Chronic Lymphocytic Leukemia. Oncol. Lett..

[104] Zahran A.M., Moeen S.M., Thabet A.F., Rayan A., Abdel-Rahim M.H., Mohamed W.M., Hetta H.F. (2020). Monocytic Myeloid-Derived Suppressor Cells in Chronic Lymphocytic Leukemia Patients: A Single Center Experience. Leuk. Lymphoma.

[105] Wilcox R.A., Feldman A.L., Wada D.A., Yang Z.Z., Comfere N.I., Dong H., Kwon E.D., Novak A.J., Markovic S.N., Pittelkow M.R. (2009). B7-H1 (PD-L1, CD274) Suppresses Host Immunity in T-Cell Lymphoproliferative Disorders. Blood.

[106] Bontkes H.J., Jordanova E.S., Nijeboer P., Neefjes-Borst E.A., Cillessen S.A., Hayat A., Mulder C.J., Bouma G., Von Blomberg B.M., De Gruijl T.D. (2017). High Myeloid-Derived Suppressor Cell Frequencies in the Duodenum Are Associated with Enteropathy Associated T-Cell Lymphoma and Its Precursor Lesions. Br. J. Haematol..

[107] Enblad G., Karlsson H., Gammelgård G., Wenthe J., Lövgren T., Amini R.M., Wikstrom K.I., Essand M., Savoldo B., Hallböök H. (2018). A Phase I/IIa Trial Using CD19-Targeted Third-Generation CAR T Cells for Lymphoma and Leukemia. Clin. Cancer Res..

[108] Grosser R., Cherkassky L., Chintala N., Adusumilli P.S. (2019). Combination Immunotherapy with CAR T Cells and Checkpoint Blockade for the Treatment of Solid Tumors. Cancer Cell.

[109] D’Aveni M., Notarantonio A.B., Bertrand A., Boulangé L., Pochon C., Rubio M.T. (2020). Myeloid-Derived Suppressor Cells in the Context of Allogeneic Hematopoietic Stem Cell Transplantation. Front. Immunol..

